# S4-4 The Wales Physical Activity Partnership during covid-19: a case study of a long-term strategic cross-sector alliance

**DOI:** 10.1093/eurpub/ckad133.021

**Published:** 2023-09-11

**Authors:** Vasiliki Kolovou, Nicola Bolton, Diane Crone

**Affiliations:** Cardiff Metropolitan University - School of Sport & Health Sciences; Cardiff School of Management; Cardiff Metropolitan University - School of Sport & Health Sciences

## Abstract

**Purpose:**

We aimed to investigate partnership-working across sectors through the example of the Wales Physical Activity Partnership (WPAP). Three national organisations specialising in sport, environment and public health formed a strategic alliance under the Welsh Government’s direction to support the long-term innovative legislation of the Wellbeing of Future Generations (Wales) Act 2015 (WBFGA).

**Methods:**

We completed in-depth semi-structured interviews with WPAP members (n = 9, August 2021 to March 2022) involved in the leadership, management and work-stream levels of the partnership. The leadership group members were interviewed twice, with 12 months between interviews, to follow-up progress of the partnership and changes in strategic direction. The interviews were transcribed and analysed in NVivo using Braun & Clarke’s (2014) thematic analysis.

**Results:**

The participants had experiences of operating in leadership, management and working group roles within the partnership, each representing their organisation. The major themes were related to the partners sudden change in short-term priorities and the efforts to maintain momentum in line with the long-term direction of their alliance. The Covid-19 pandemic was felt to accentuate underlying issues, by limiting capacity and halting momentum in the developing the partners’ shared agenda. The leadership group remained confident of the added value to their organisations by connecting sport, environment and public health and recognised the shared responsibility of their organisation in supporting the Welsh Government to increase physical activity across Wales. The working group members felt that the governance structure and division of work-streams diluted the potential of the three partners, and often felt “compartmentalised” and there was limited communication between work-streams.

**Conclusions:**

The impact from the Covid-19 pandemic was a barrier to building and maintaining momentum in this partnership between sport environment and public health. Wales is the first country to impose that public organisations bear a legislative duty to evidence their contribution towards the long-term strategy to improve physical activity, health and wellbeing via the WBFGA. Innovative legislation has provided a foundation for organisations in diverse sectors to shift their agendas to evidence long-term contributions but short-term pressures, such as the Covid-19 pandemic, may dilute the commitment and strategic intent.

